# RCCD1-JMJD5 coordinate histone demethylation to promote platinum resistance in non-small cell lung cancer via FN1-mediated epithelial–mesenchymal transition

**DOI:** 10.1186/s43556-026-00625-y

**Published:** 2026-10-21

**Authors:** Lili Tan, Yinghui Tong, Wanying Zhou, Xinyue Ma, Qilu Fang, Yan Feng, Xueping Xiang, Luo Fang

**Affiliations:** 1 Zhejiang Cancer Hospital, Hangzhou Institute of Medicine (HIM), Chinese Academy of Sciences, Hangzhou, 310022 Zhejiang China; 2 Postgraduate·Training Base Alliance of Wenzhou Medical University·(Zhejiang Cancer Hospital), Hangzhou, 310022 Zhejiang China; 3 Department of Pathology, the Second Affiliated Hospital, School of Medicine, Zhejiang University, Hangzhou, 310009 China

**Keywords:** Non-small cell lung cancer, JMJD5, RCCD1, H3K36me2, Fibronectin 1, Platinum resistance

## Abstract

Chemoresistance remains a major obstacle to effective treatment of non-small cell lung cancer (NSCLC), yet the epigenetic mechanisms underlying platinum resistance remain incompletely understood. Here, we show that Jumonji C domain-containing protein 5 (JMJD5) promotes platinum resistance in NSCLC cells and xenograft tumors while inducing a slow-proliferative, mesenchymal-like phenotype characterized by enhanced migration, invasion, spheroid formation, and epithelial–mesenchymal transition (EMT). Mechanistically, JMJD5 interacted with RCC1 domain-containing protein 1 (RCCD1), and cisplatin treatment promoted their nuclear accumulation concomitant with reduced H3K36me2 levels. RCCD1 depletion attenuated JMJD5-associated H3K36me2 demethylation and reversed EMT-associated phenotypes and cisplatin resistance, supporting RCCD1 as a functional cofactor of JMJD5-mediated chromatin remodeling. Fibronectin 1 (FN1) was further identified as a downstream effector of this pathway. Genetic or pharmacological inhibition of FN1 suppressed JMJD5-induced migration and invasion and restored cisplatin sensitivity. Notably, JMJD5-dependent FN1 protein accumulation required ongoing protein synthesis without a corresponding increase in FN1 mRNA, suggesting post-transcriptional regulation. Consistently, elevated FN1 expression was associated with unfavorable overall survival in patients with lung cancer. Collectively, these findings reveal a JMJD5–RCCD1–H3K36me2–FN1 regulatory axis linking chemotherapy-induced epigenetic remodeling to EMT-associated platinum resistance and suggest that disruption of this pathway may represent a potential strategy for overcoming chemoresistance in NSCLC.

**Supplementary Information:**

The online version contains supplementary material available at https://doi.org/10.1186/s43556-026-00625-y.

## Introduction

Lung cancer is one of the most prevalent malignancies worldwide and the leading cause of cancer-related mortality [1, 2]. Non-small cell lung cancer (NSCLC) accounts for approximately 80–85% of all cases [3]. In recent years, the management of NSCLC has been revolutionized by the advent of novel therapeutic approaches, leading to substantial improvements in survival outcomes. Nevertheless, chemotherapy remains a critical treatment option for NSCLC patients in various clinical scenarios, including, but not limited to, its use in combination with immune checkpoint inhibitors or for patients lacking oncogenic drivers. Elucidating the molecular mechanisms and key mediators of chemoresistance is therefore essential for developing novel therapeutic strategies. In a prior study [4], we demonstrated that elevated expression of JMJD5 (Jumonji domain-containing protein 5) in NSCLC tumor tissues was associated with significantly worse prognosis in patients receiving platinum-based chemotherapy drugs, suggesting a potential role for JMJD5 in mediating chemoresistance. However, the precise mechanisms underlying this association remain unclear. Thus, here we conducted the present study to delineate the functional role of JMJD5 in mediating chemoresistance in NSCLC.

As a member of the JmjC family, JMJD5, also known as lysine demethylase 8 (KDM8), has been reported to exhibit multiple epigenetic regulatory functions. Initially, JMJD5 was identified as a histone H3K36me2 demethylase [5]. Subsequently, studies have reported that JMJD5 exhibits arginyl C-3 hydroxylase [6] and protease activities [7–9] that cleave specific histone tails. Liu et al. [7] found that CDK9 phosphorylates the C-terminal region of Pol II and promotes the recruitment of JMJD5 to Pol II. This recruitment facilitates JMJD5 entry into + 1 nucleosomes, generating tailless nucleosomes and enhancing gene transcription. Furthermore, JMJD5 specifically cleaves histone 2,3,4 tails containing methylated arginine and acts as an aminopeptidase to digest the C-terminal product [8]. Under DNA-damaging stress, JMJD5 acts as a Cathepsin L-type protease and mediates hydrolytic cleavage of histone H3 N-tail proteins [9], thereby contributing to chromatin remodeling. Given these multifunctional epigenetic activities, we hypothesize that JMJD5-mediated chemoresistance development may rely on its epigenetic modification capabilities.

It is noteworthy that JMJD5 may exert distinct roles in different tumor contexts, such as suppressing tumor progression or promoting drug resistance. For instance, JMJD5 positively regulates CDKN1A transcription, thereby inhibiting cell proliferation of hepatocellular carcinoma [10]. Similarly, in pancreatic cancer cells, JMJD5 negatively regulates the expression of c-Myc, thereby inhibiting cell proliferation and aerobic glycolysis [11]. However, in prostate cancer, JMJD5 coactivates AR and PKM2 to mediate tumor metabolism and enzalutamide resistance [12]. Notably, in some cases, JMJD5 can perform divergent functions through interactions with different proteins. For instance, it has been found that JMJD5 promotes the dimerization of pyruvate kinase M2 (PKM2), thereby inhibiting its enzymatic activity [13]. It also binds to PKP2 to regulate its nuclear translocation and acts as a coactivator of HIF-1α to upregulate glycolytic genes [14]. Furthermore, JMJD5 regulates the cell cycle, cell proliferation, and metastasis through its interaction with p53 [15, 16]. A recent study reported that JMJD5 binds to the epidermal growth factor receptor (EGFR) and promotes its degradation in lung cancer [15]. In summary, JMJD5 plays diverse roles in cancer and often relies on interacting partners for its function. Therefore, in this study, we also explored proteins that can bind to JMJD5 and investigated their roles in JMJD5-mediated chemoresistance in NSCLC.

Despite increasing evidence implicating JMJD5 in tumor progression, the molecular mechanisms by which it mediates cisplatin resistance remain poorly understood. In the present study, we investigated the epigenetic basis of JMJD5-driven chemoresistance in NSCLC using in vitro and in vivo models. Our findings identify RCCD1 as a critical cofactor required for JMJD5-mediated H3K36me2 demethylation and reveal FN1 as a key downstream effector linking epigenetic reprogramming to mesenchymal transition and chemoresistance. These results provide mechanistic insight into the epigenetic regulation of cisplatin resistance and highlight the JMJD5-RCCD1-FN1 signaling axis as a promising therapeutic vulnerability for sensitizing NSCLC to chemotherapy.

## Results

### *JMJD5 promotes chemoresistance in NSCLC cells *in vivo* and *in vitro

**Fig. 1:**
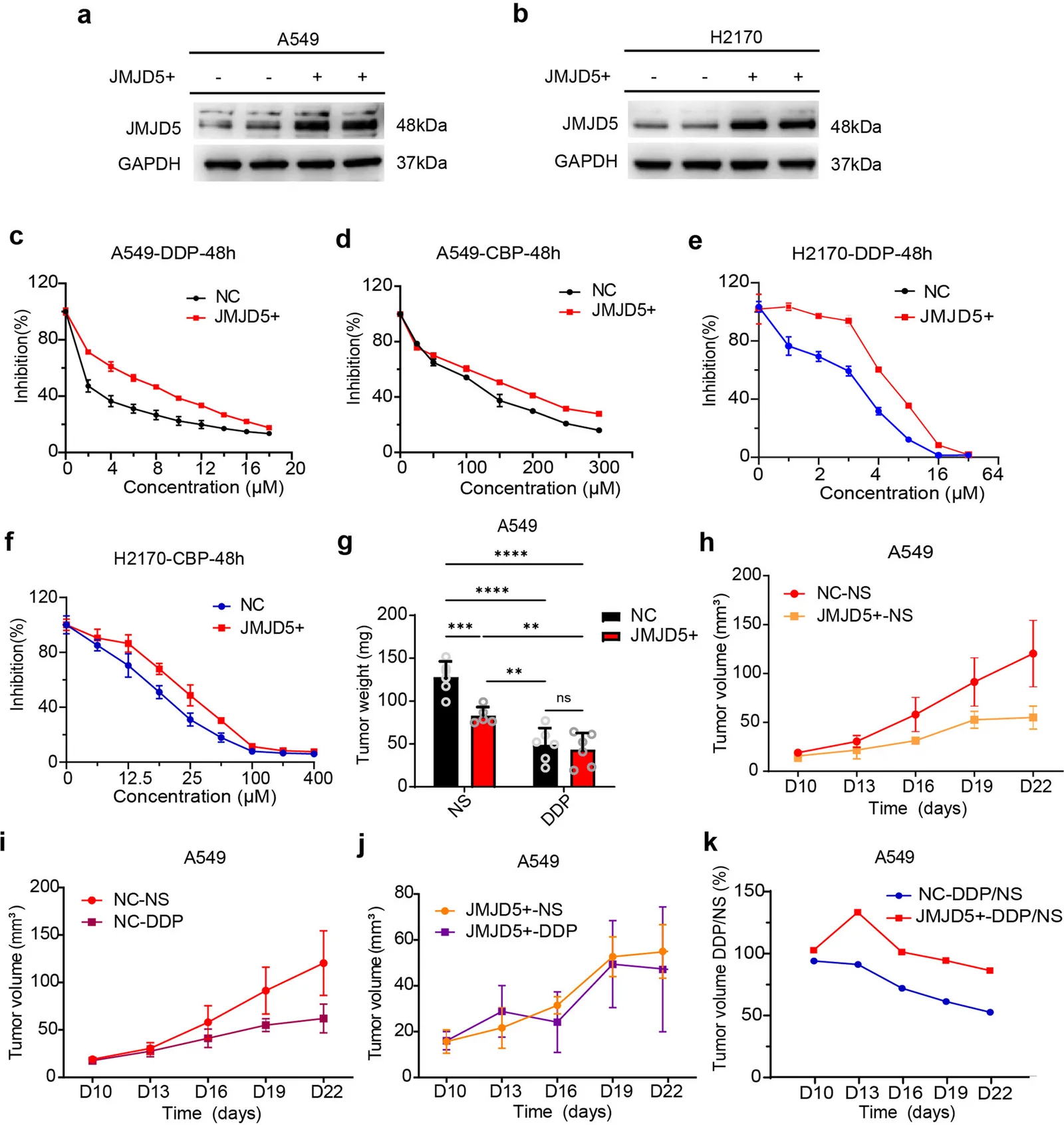
JMJD5 promotes chemoresistance in NSCLC cells. **a**, **b** Western blot analysis confirming stable JMJD5 overexpression in A549 (**a**) and H2170 (**b**) cells. **c**, **d** Drug sensitivity of A549^NC^ and A549^JMJD5+^ cells to cisplatin (**c**) and carboplatin (**d**). **e**, **f** Drug sensitivity of H2170^NC^ and H2170^JMJD5+^ cells to cisplatin (**e**) and carboplatin (**f**). **g** Tumor weights of A549^NC^ and A549^JMJD5+^-derived xenografts at the experimental endpoint. **h** Growth curves of subcutaneous xenografts derived from A549^NC^ and A549^JMJD5+^ cells in BALB/c nude mice. Tumor volume was measured every 3 days and calculated as (length × width^2^)/2. Each xenograft group included 12 mice, with six mice receiving cisplatin (DDP; 6 mg/kg) and six receiving normal saline (NS). **i**, **j** Growth curves of A549^NC^ (**i**) and A549^JMJD5+^ (**j**) xenografts treated with DDP or NS. Treatment was initiated when tumor volumes reached 100–200 mm^3^ and administered via tail-vein injection every 3 days. Mice were sacrificed 12 days after the first treatment for tumor collection and weighing. **k** Chemosensitivity of A549^NC^ and A549.^JMJD5+^ xenografts, expressed as the ratio of tumor volume in the DDP-treated group to that in the corresponding NS-treated group. NC, negative control; JMJD5 +, JMJD5-overexpressing cells; DDP, cisplatin; NS, normal saline. Day 0 indicates the day of treatment initiation rather than the day of tumor-cell inoculation. Data are presented as mean ± SD. *P* < 0.05 was considered statistically significant; ns, not significant; *P < 0.05, **P < 0.01, ***P < 0.001 and ****P < 0.0001

A previous study from our team demonstrated that high JMJD5 expression is associated with poor survival prognosis in NSCLC patients after chemotherapy [4]. To investigate the underlying mechanism of this association, we generated NSCLC cell lines stably overexpressing JMJD5 (A549^JMJD5+^ and H2170^JMJD5+^) using lentiviral infection (Fig. 1a-b). Then, we evaluated the sensitivity of JMJD5 stably overexpressing NSCLC cell lines to chemotherapeutic drugs commonly used for NSCLC in vitro. The data revealed that elevated JMJD5 expression reduced sensitivity to cisplatin (DDP) and carboplatin (CBP) compared to the control group (Fig. 1c-f). However, JMJD5 overexpression did not confer resistance to other chemotherapeutic agents, including pemetrexed (PBM) and paclitaxel (PTX) (Fig. S1a-b). This suggests that JMJD5 confers resistance to platinum-based chemotherapy for non-small cell lung cancer.

The xenograft experiment was also performed to examine the influence of JMJD5 overexpression on chemotherapy response in vivo. A549^NC^ and A549^JMJD5+^ cells were subcutaneously inoculated into BALB/c nude mice. The results showed that JMJD5 overexpression exhibited slower tumor growth (Fig. 1g-h, Fig. S1c-e) and increased chemoresistance (Fig. 1i-j). The tumor growth inhibition by cisplatin (DDP), calculated based on tumor volume, was significantly lower in the JMJD5-overexpressing group (A549^JMJD5+)^ than in the control group (A549^NC^)(Fig. 1k). These findings indicate that JMJD5 overexpression enhances chemoresistance in A549 cells. Interestingly, our research findings reveal that JMJD5 upregulation inhibits non-small cell lung cancer growth while promoting platinum-based chemotherapy resistance. This contradictory outcome raises critical questions about JMJD5's clinical relevance. To address this, we further investigated the key mechanisms by which JMJD5 contributes to platinum resistance in non-small cell lung cancer.

### JMJD5 inhibits the proliferation of NSCLC cells

**Fig. 2:**
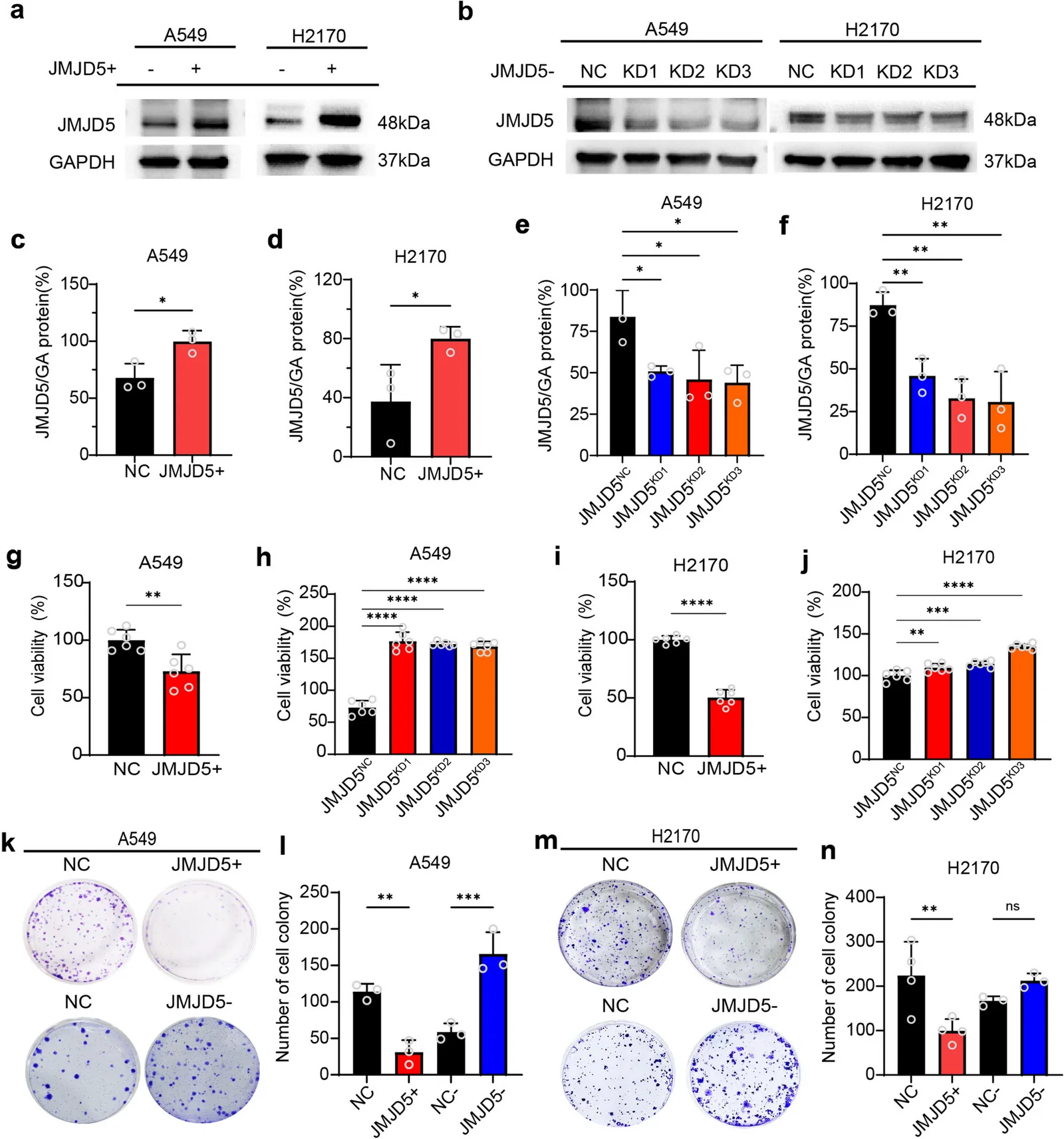
JMJD5 suppresses proliferation and clonogenic growth of A549 and H2170 cells. **a** Western blot analysis confirming stable JMJD5 overexpression in A549 and H2170 cells. **b** Western blot analysis confirming JMJD5 knockdown in A549 and H2170 cells. **c**, **d**, **e**, **f** Densitometric quantification of JMJD5 protein levels normalized to GAPDH in A549 (**c**, **e**) and H2170 (**d**, **f**) cells following JMJD5 overexpression or knockdown. **g**, **h** CCK-8 assays showing changes in A549 cell proliferation following JMJD5 overexpression (g) or knockdown (h). **i**, **j** CCK-8 assays showing changes in H2170 cell proliferation following JMJD5 overexpression (i) or knockdown (j). **k, l** Colony formation assays evaluating the clonogenic capacity of A549 cells following JMJD5 overexpression or knockdown. **m**, **n** Colony formation assays evaluating the clonogenic capacity of H2170 cells following JMJD5 overexpression or knockdown. NC, negative control; JMJD5 +, JMJD5-overexpressing cells; JMJD5 −, JMJD5-knockdown cells. Data are presented as mean ± SD from at least three biologically independent experiments. *P < 0.05, **P < 0.01, ***P < 0.001 and ****P < 0.0001

First, we employed CRISPR/Cas9 technology to achieve JMJD5 gene knockout using the pLenti-U6-sgRNA-SFFV-Cas9-2A-Puro vector. For JMJD5 overexpression, a FLAG-tagged JMJD5 construct was delivered via a lentiviral vector based on the pLenti-CMV-CBH-Puro Amp backbone. Subsequently, we investigated its effects on cell growth in vitro using CCK-8 and colony formation assays with established A549 and H2170 cells with JMJD5 overexpression or knockdown (Fig. 2a-f, *P* < 0.05). The CCK-8 assay results indicated that JMJD5 overexpression significantly inhibited cell proliferation in both A549 cells (Fig. 2g, *P* < 0.05) and H2170 cells (Fig. 2i, *P* < 0.05), whereas JMJD5 knockdown enhanced proliferation in these cell lines (Fig. 2h, j, *P* < 0.05). The colony formation assay showed that JMJD5 overexpression also reduced clonogenic capacity in A549 cells (Fig. 2k-l, *P* < 0.05) and H2170 cells (Fig. 2m-n, *P* < 0.05). These results collectively demonstrate that JMJD5 inhibits the proliferation of NSCLC cells.

### JMJD5 promotes cell migration and invasion, EMT and tumor spheroid formation in NSCLC cells

**Fig. 3:**
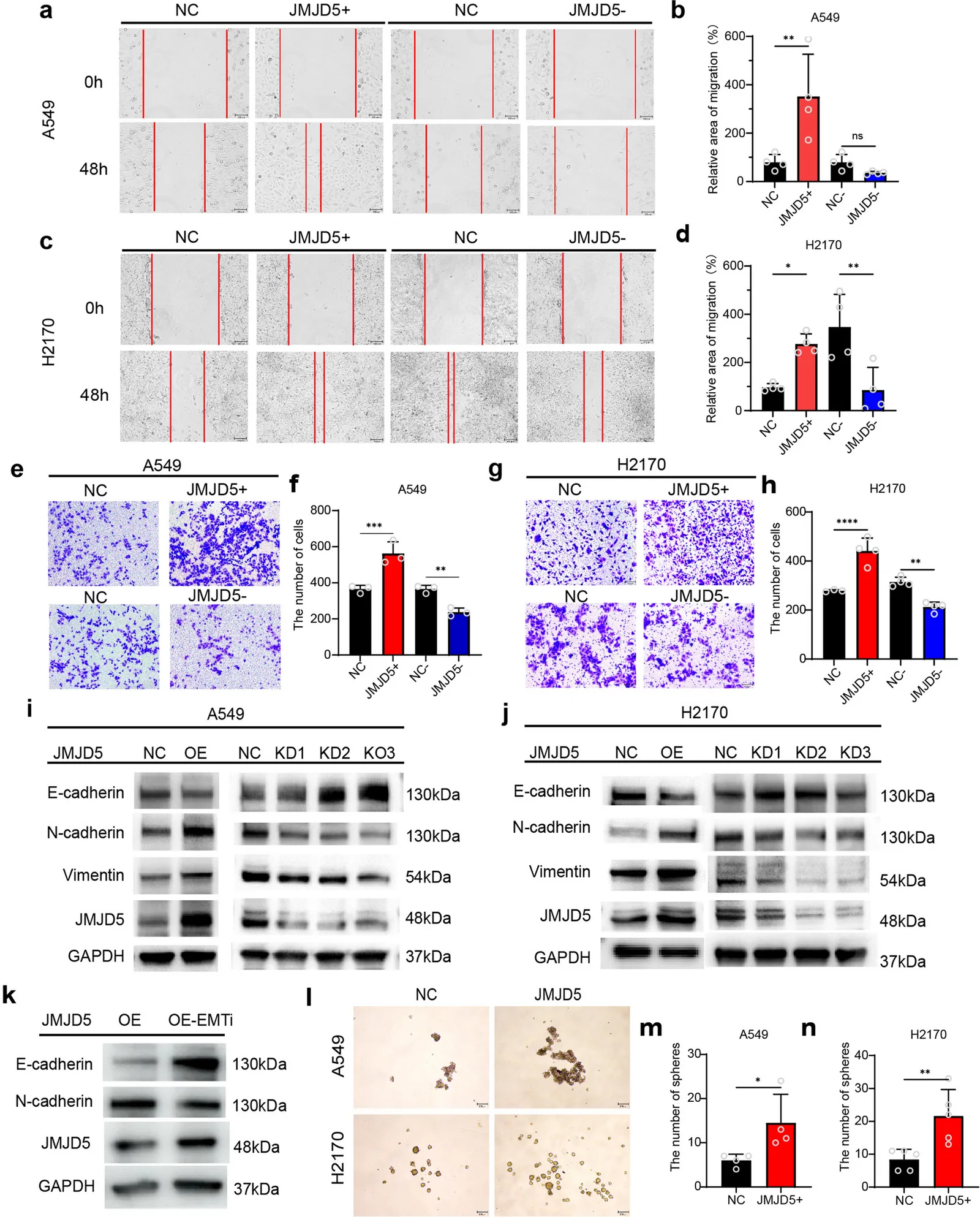
JMJD5 promotes EMT-associated malignant phenotypes in NSCLC cells.** a**, **b** Wound-healing assay assessing the migratory capacity of A549 cells following JMJD5 overexpression or knockdown (**a**), with quantitative analysis of the relative migrated area (**b**), scale bar, 100 μm. **c**, **d** Wound-healing assay assessing the migratory capacity of H2170 cells following JMJD5 overexpression or knockdown (**c**), with quantitative analysis of the relative migrated area (**d**), scale bar, 100 μm. **e**, **f** Transwell invasion assay of A549 cells following JMJD5 overexpression or knockdown (**e**), with quantification of invaded cells (**f**), scale bar, 100 μm. **g**, **h** Transwell invasion assay of H2170 cells following JMJD5 overexpression or knockdown (**g**), with quantification of invaded cells (**h**), scale bar, 100 μm. **i**, **j** Western blot analysis of EMT-related proteins in A549 (**i**) and H2170 (**j**) cells following JMJD5 overexpression or knockdown. **k** Western blot analysis of EMT-related proteins in monoclonally selected A549 cells with stable JMJD5 overexpression following treatment with an EMT inhibitor. **l** Representative images of tumor spheroids formed by A549 and H2170 cells with different JMJD5 expression levels. Scale bar, 100 μm. **m**, **n** Quantification of spheroid numbers in A549 (m) and H2170 (n) cells. Data are presented as mean ± SD from at least three biologically independent experiments. *P < 0.05, **P < 0.01, ***P < 0.001 and ****P < 0.0001

The results of the wound-healing assay showed that JMJD5 overexpression significantly enhanced cell migration in both A549 (Fig. 3a-b, *P* < 0.05) and H2170 cells (Fig. 3c-d, *P* < 0.05), whereas JMJD5 knockdown markedly attenuated migratory ability in these cell lines. Transwell assay further revealed that JMJD5 overexpression in A549 cells (Fig. 3e-f, *P* < 0.05) and H2170 cells (Fig. 3g-h, *P* < 0.05) significantly promoted cell invasion, while JMJD5 knockdown exerted the opposite effect.

Subsequently, we examined the expression of epithelial-mesenchymal transition (EMT) marker proteins by Western blot in A549 cells and H2170 cells with different JMJD5 expression levels. The results showed that overexpression of JMJD5 decreased the expression of the epithelial marker E-cadherin and increased the expression of the mesenchymal marker N-cadherin and vimentin in A549 (Fig. 3i) cells and H2170 (Fig. 3j) cells. This trend was reversed when JMJD5 was downregulated, suggesting that JMJD5 can promote the occurrence of EMT molecular phenotypic changes in NSCLC cells.

To further elucidate the functional role of JMJD5 in mediating the malignant phenotype of EMT, we treated monoclonally selected, stably overexpressing A549 cells expressing JMJD5 with an EMT-specific inhibitor (EMTi). The results demonstrated that EMT treatment significantly reversed the EMT phenotype induced by JMJD5 overexpression, characterized by reduced N-cadherin expression and markedly restored E-cadherin expression. Notably, EMT treatment did not affect the expression level of JMJD5 protein (Fig. 3k). These findings indicate that JMJD5 resides upstream of the EMT program; its promotion of EMT does not arise from feedback regulation of JMJD5 by EMT-related proteins but rather through activation of the EMT pathway to induce epithelial-mesenchymal transition in NSCLC cells, further supporting EMT as a critical downstream mechanism through which JMJD5 exerts its pro-tumor effects.

We further investigated whether JMJD5 also participates in regulating the stem cell characteristics of lung cancer cells. The results showed that JMJD5 overexpression in A549 and H2170 cells significantly increased the number of tumor spheroids (Fig. 3l-n, *P* < 0.05). These results suggest that JMJD5 overexpression significantly enhanced the tumor stem cell-like properties of the cells. Collectively, our results indicate that JMJD5 overexpression significantly enhances mesenchymal-like properties, including increased migration ability, epithelial-mesenchymal transition (EMT)-associated traits, and stemness characteristics.

Based on our proliferation assays and chemoresistance analyses, we speculate that JMJD5 may contribute to platinum resistance in NSCLC by promoting a stem-like or mesenchymal-like phenotype, characterized by enhanced migratory and invasive capabilities. The observed reduction in proliferation is likely a consequence of the acquisition of these resistant traits.

### RCCD1 is a key chaperone of JMJD5-mediated histone H3K36me2 demethylation

**Fig. 4:**
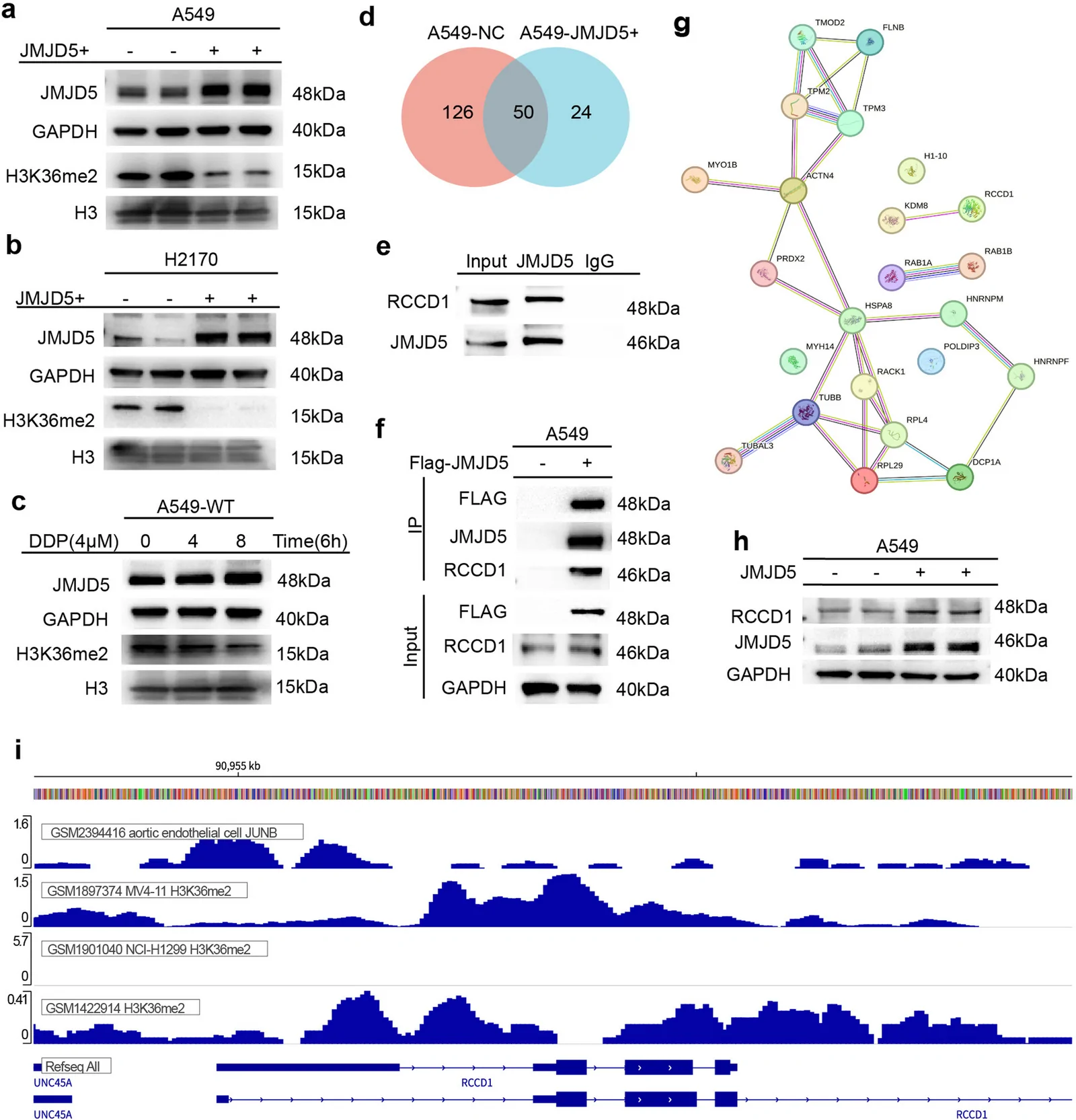
RCCD1 interacts with JMJD5 and is associated with JMJD5-mediated H3K36me2 demethylation. **a**, **b** Western blot analysis showing enhanced H3K36me2 demethylation following JMJD5 overexpression in A549 (**a**) and H2170 (**b**) cells. **c** Western blot analysis showing increased JMJD5 expression and enhanced H3K36me2 demethylation in A549 cells following cisplatin (DDP) treatment. **d** Immunoprecipitation–mass spectrometry (IP–MS) analysis for identification of JMJD5-interacting proteins. The Venn diagram shows overlapping differentially expressed proteins identified between A549^JMJD5+^ and A549^NC^ groups. **e**, **f** Co-immunoprecipitation assays confirming the interaction between JMJD5 and RCCD1. **g** STRING database analysis identifying RCCD1 as a potential JMJD5-interacting protein. **h** Western blot analysis showing increased RCCD1 expression following JMJD5 upregulation. **i** Public ChIP-seq data from the Cistrome database showing H3K36me2 enrichment at the RCCD1 genomic locus. NC, negative control; JMJD5 +, JMJD5-overexpressing cells; DDP, cisplatin

JMJD5 was previously reported as a histone demethylase, which was verified in our study. In A549 and H2170 cells, overexpression of JMJD5 resulted in increased demethylation of H3K36me2 (Fig. 4a-b). We then investigated whether the regulation of chemoresistance by JMJD5 is related to its demethylase activity. Our results showed that DDP stimulation promoted JMJD5 expression, which was accompanied by increased H3K36 demethylation(Fig. 4c, Fig. S1f-g). Since JMJD5 lacks a recognizable histone-binding domain [17], we hypothesized that a chaperone protein may be required for its function as a histone demethylase.

Therefore, we performed co-immunoprecipitation (Co-IP) coupled with mass spectrometry (MS) to identify potential JMJD5-binding proteins. Comparative analysis revealed 24 proteins with significantly altered expression levels in A549^JMJD5+^ cells compared to A549^NC^ cells, including JMJD5 itself (Fig. 4d). These candidate interactions were further validated through Co-IP using anti-JMJD5 antibody (Supplementary Fig. 1 h). Notably, RCCD1 was identified as the chaperone demonstrating endogenous interaction with JMJD5 (Fig. 4e), as confirmed by flag-tagged pull-down assays in A549^JMJD5+^ cells (Fig. 4f). Similarly, the JMJD5-RCCD1 interaction was also predicted by the STRING database (Fig. 4g). Interestingly, our analysis revealed that RCCD1 expression is positively correlated with JMJD5 expression (Fig. 4h), suggesting a potential functional relationship between the two proteins.

To further investigate whether JMJD5 overexpression–induced alterations in H3K36me2 are associated with RCCD1, we analyzed publicly available H3K36me2 ChIP-seq datasets from the Cistrome Data Browser. Across multiple independent datasets, a consistent enrichment of H3K36me2 peaks was observed at the RCCD1 genomic locus (Fig. 4i), suggesting a potential association between RCCD1 and H3K36me2 histone modification. Consistent with our experimental findings that JMJD5 overexpression upregulates RCCD1 expression while concomitantly reducing global H3K36me2 levels, these results support a model in which JMJD5 may regulate H3K36me2-mediated chromatin states in part through modulation of RCCD1 expression. This JMJD5–RCCD1 axis may, in turn, contribute to the transcriptional regulation of downstream target genes via H3K36me2-dependent epigenetic remodeling.

### RCCD1 is required for JMJD5-mediated demethylase activity and the promotion of mesenchymal-like phenotype and chemoresistance upon DDP treatment

**Fig. 5:**
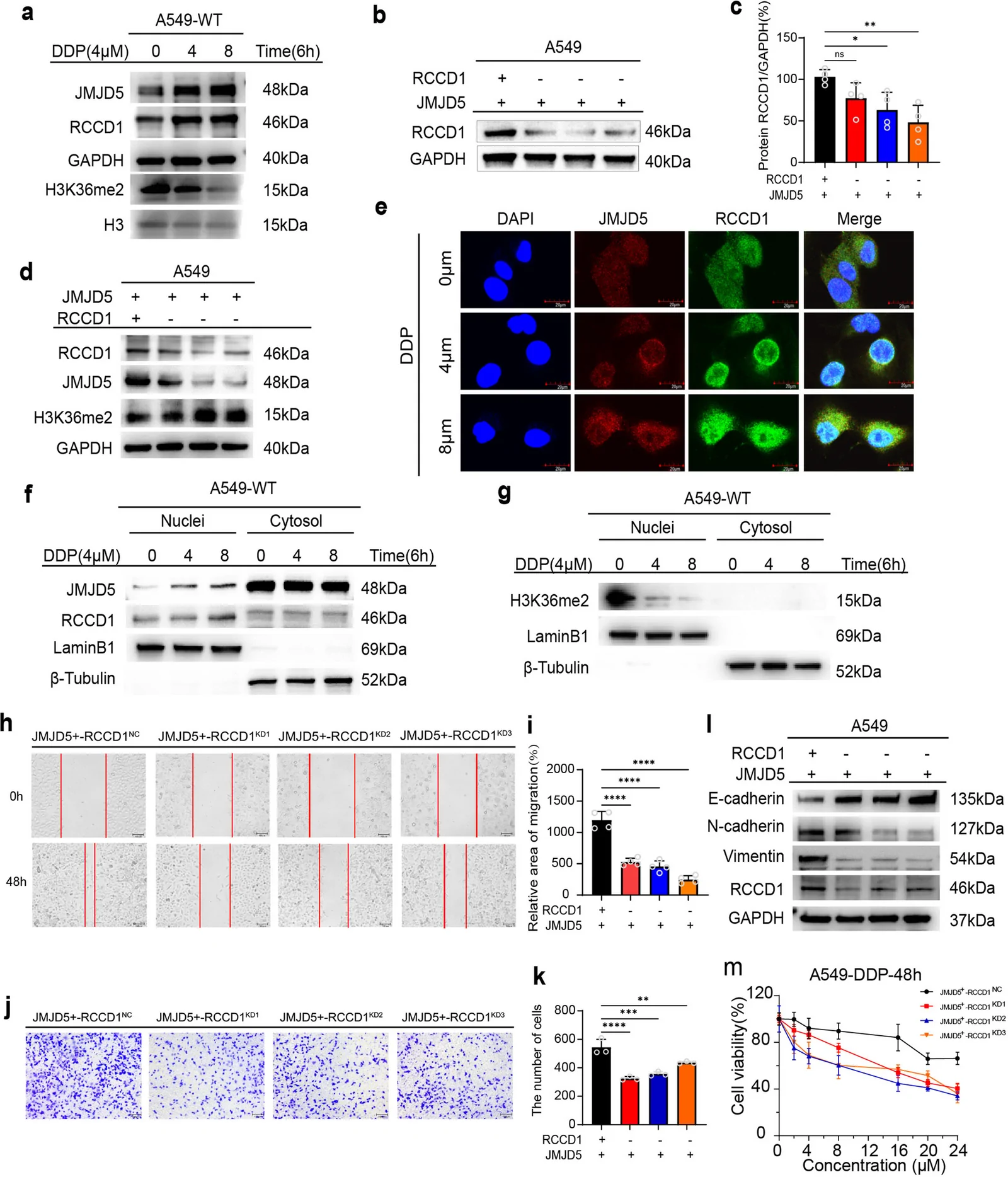
JMJD5 mediates H3K36me2 demethylation under cisplatin-induced stress in an RCCD1-dependent manner. **a** Western blot analysis showing that cisplatin (DDP) treatment increased JMJD5 and RCCD1 expression and enhanced H3K36me2 demethylation in A549 cells. **b**, **c** Western blot analysis of RCCD1 knockdown efficiency in A549^JMJD5+^ cells using siRNA (**b**), with densitometric quantification of RCCD1 protein levels normalized to GAPDH (**c**). **d** Western blot analysis showing that RCCD1 knockdown attenuated JMJD5 overexpression-induced H3K36me2 demethylation. **e** Confocal microscopy showing the subcellular localization and fluorescence intensity of JMJD5 and RCCD1 in A549 cells following DDP treatment for 6 h (60 × oil-immersion objective). **f**, **g** Western blot analysis of the subcellular distribution of JMJD5 and RCCD1 and H3K36me2 demethylation in A549 cells following 6 h of DDP treatment. **h**, **i** Wound-healing assay showing that RCCD1 knockdown attenuated the enhanced migratory capacity of A549^JMJD5+^ cells (**h**), with quantification of the relative migrated area (**i**), Scale bar, 100 μm. **j**, **k** Transwell invasion assay showing that RCCD1 knockdown suppressed JMJD5-induced cell invasion (**j**), with quantification of invaded cells (**k**), Scale bar, 100 μm. **l** Western blot analysis of EMT-related proteins in A549^JMJD5+^ cells following RCCD1 knockdown. m CCK-8 assay evaluating cisplatin sensitivity of A549.^JMJD5+^ cells following RCCD1 knockdown. JMJD5 +, JMJD5-overexpressing cells; DDP, cisplatin. All experiments were repeated three times. Data are presented as mean ± SD. *P < 0.05, **P < 0.01, ***P < 0.001 and ****P < 0.0001

We next investigated RCCD1's role in JMJD5-mediated histone demethylation. Notably, DDP stimulation induced RCCD1 upregulation in A549 cells (Fig. 5a, Fig. S2a), which paralleled the previously characterized JMJD5 elevation and associated H3K36me2 demethylation. We then established RCCD1-knockdown cells in JMJD5-overexpressing A549 cells using shRNA (designated as A549^JMJD5+^-RCCD1^NC^, A549^JMJD5+^-RCCD1^KD1^, A549^JMJD5+^-RCCD1^KD2^, and A549^JMJD5+^-RCCD1^KD3^) (Fig. 5b-c). RCCD1 knockdown reversed H3K36me2 demethylation promoted by JMJD5 overexpression(Fig. 5d), suggesting that RCCD1 acts as a key chaperone of JMJD5-mediated histone H3K36me2 demethylation. Meanwhile, in A549 cells after cisplatin treatment, immunofluorescence (Fig. 5e) and Western blot (Fig. 5f, Fig. S2b-c) analyses revealed increased nuclear translocation of both JMJD5 and RCCD1 accompanied by H3K36me2 demethylation (Fig. 5g, Fig. S2d), the nuclear H3K36me2 level significantly decreased after treatment with 8 μM DDP (*P* < 0.05). This suggests that the JMJD5-RCCD1 complex may mediate the response to chemotherapy-induced stress through histone H3K36me2 demethylation.

Subsequently, we explored the role of RCCD1 in JMJD5 regulation of the mesenchymal-like phenotype. The data showed that RCCD1 knockdown significantly reversed cell migration (Fig. 5h-i, *P* < 0.05), invasion (Fig. 5j-k, *P* < 0.05) and epithelial-mesenchymal transition (EMT) (Fig. 5l) promoted by JMJD5 overexpression. In addition, RCCD1 knockdown also reversed cisplatin resistance induced by JMJD5 in A549 cells (Fig. 5m). These results comprehensively demonstrate that RCCD1 plays an important role in JMJD5-regulated mesenchymal-like phenotype and chemoresistance.

### Fibronectin 1 (FN1) is a potential downstream molecule of JMJD5 for EMT regulation

**Fig. 6:**
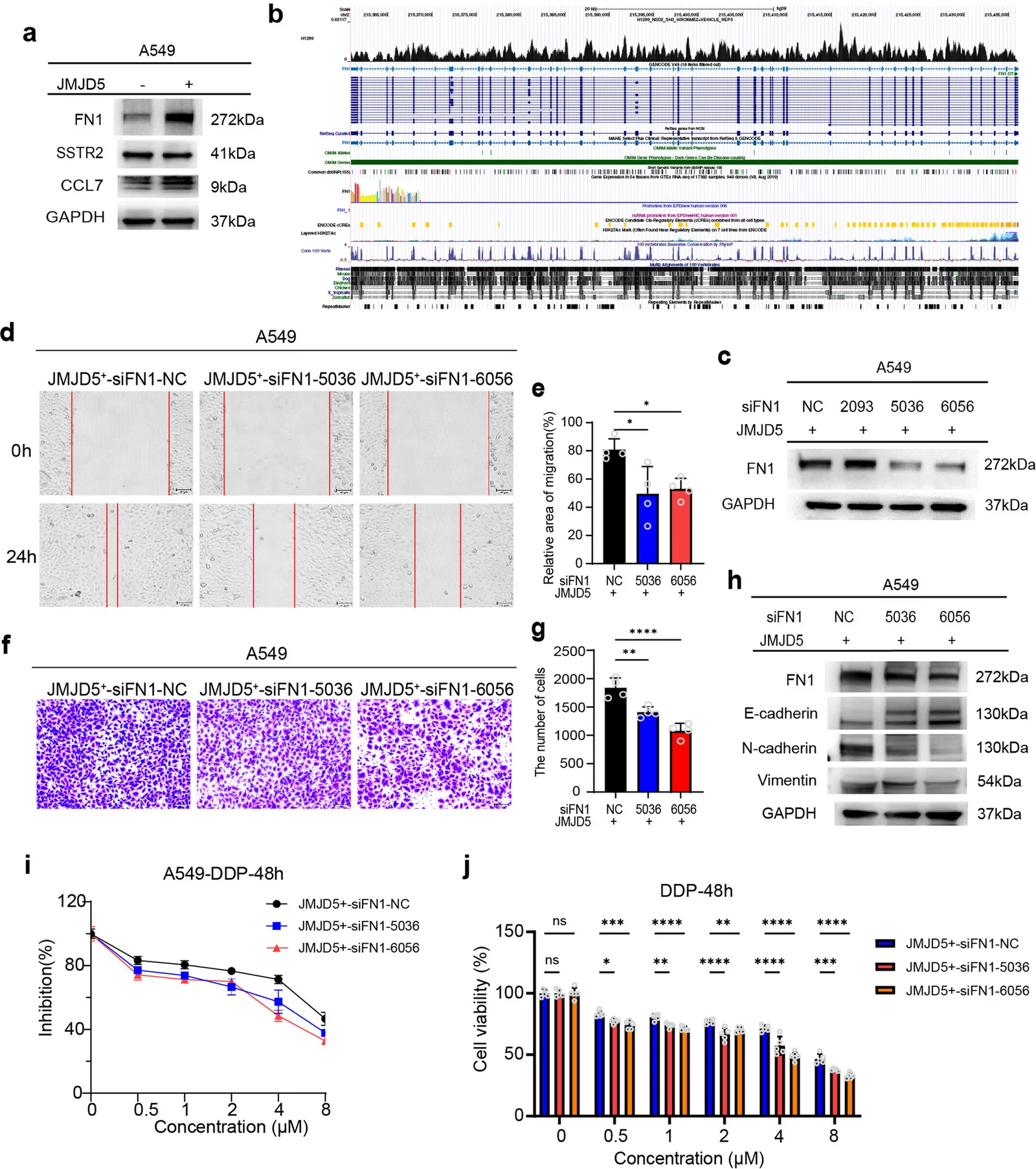
FN1 acts as a downstream effector of JMJD5 to regulate EMT-associated malignant phenotypes and cisplatin resistance. **a** Western blot analysis of candidate downstream proteins following JMJD5 overexpression. **b** Cistrome DB analysis of publicly available ChIP-seq data showing H3K36me2 enrichment at the FN1 genomic locus. c Western blot analysis confirming siRNA-mediated FN1 knockdown in A549^JMJD5+^ cells. **d**, **e** Wound-healing assay showing reduced migration of A549^JMJD5+^ cells following FN1 knockdown (**d**), with quantitative analysis of the relative migrated area (**e**), Scale bar, 100 μm. **f**, **g** Transwell invasion assay showing reduced invasive capacity of A549^JMJD5+^ cells following FN1 knockdown (**f**), with quantification of invaded cells (**g**), Scale bar, 100 μm. **h** Western blot analysis of EMT-related proteins in A549^JMJD5+^ cells following FN1 knockdown. **i**, **j** CCK-8 assay evaluating the effect of FN1 knockdown on cisplatin sensitivity in A549.^JMJD5+^ cells (**i**), with statistical analysis of the treatment response (**j**). JMJD5 +, JMJD5-overexpressing cells. siFN1-5036 and siFN1-6056 represent two independent siRNA sequences targeting FN1. All experiments were repeated three times. Data are presented as mean ± SD. *P < 0.05, **P < 0.01, ***P < 0.001 and ****P < 0.0001

To further investigate the mechanisms by which JMJD5 regulates mesenchymal-like phenotypes, we performed a PCR array screening for potential downstream molecules of JMJD5. The top three genes showing the most pronounced expression changes (fold change > 5) were selected for subsequent validation using Western blot and qPCR assay FN1 protein was found to be significantly increased after JMJD5 overexpression in A549 cells (Fig. 6a, Fig. S2e-f). Notably, based on ChIP-seq data from public databases, we identified significant H3K36me2 enrichment in the FN1 gene region (Fig. 6b). This suggests that FN1 may serve as a potential downstream target regulated by the combined action of JMJD5 and RCCD1 to mediate H3K36me2 demethylation.

Subsequently, we downregulated FN1 expression in A549 cells with stable JMJD5 overexpression using siRNA (Fig. 6c) to investigate whether FN1 plays a role in maintaining the mesenchymal-like phenotype. Functional analysis demonstrated that FN1 deficiency could reverse the mesenchymal-like phenotype induced by JMJD5 overexpression: reduced cell migration capacity (Fig. 6d-e, *P* < 0.05), diminished invasive ability (Fig. 6f-g, *P* < 0.05), and reversed epithelial-mesenchymal transition (EMT) phenomena (Fig. 6h). Additionally, FN1 knockdown reversed cisplatin resistance induced by JMJD5 in A549 cells (Fig. 6i-j, *P* < 0.05). These results suggest that FN1 may serve as a key downstream target for JMJD5 in regulating the mesenchymal-like phenotype and drug resistance in NSCLC.

### FN1 mediates JMJD5-driven cisplatin resistance and is associated with poor prognosis in lung cancer

**Fig. 7:**
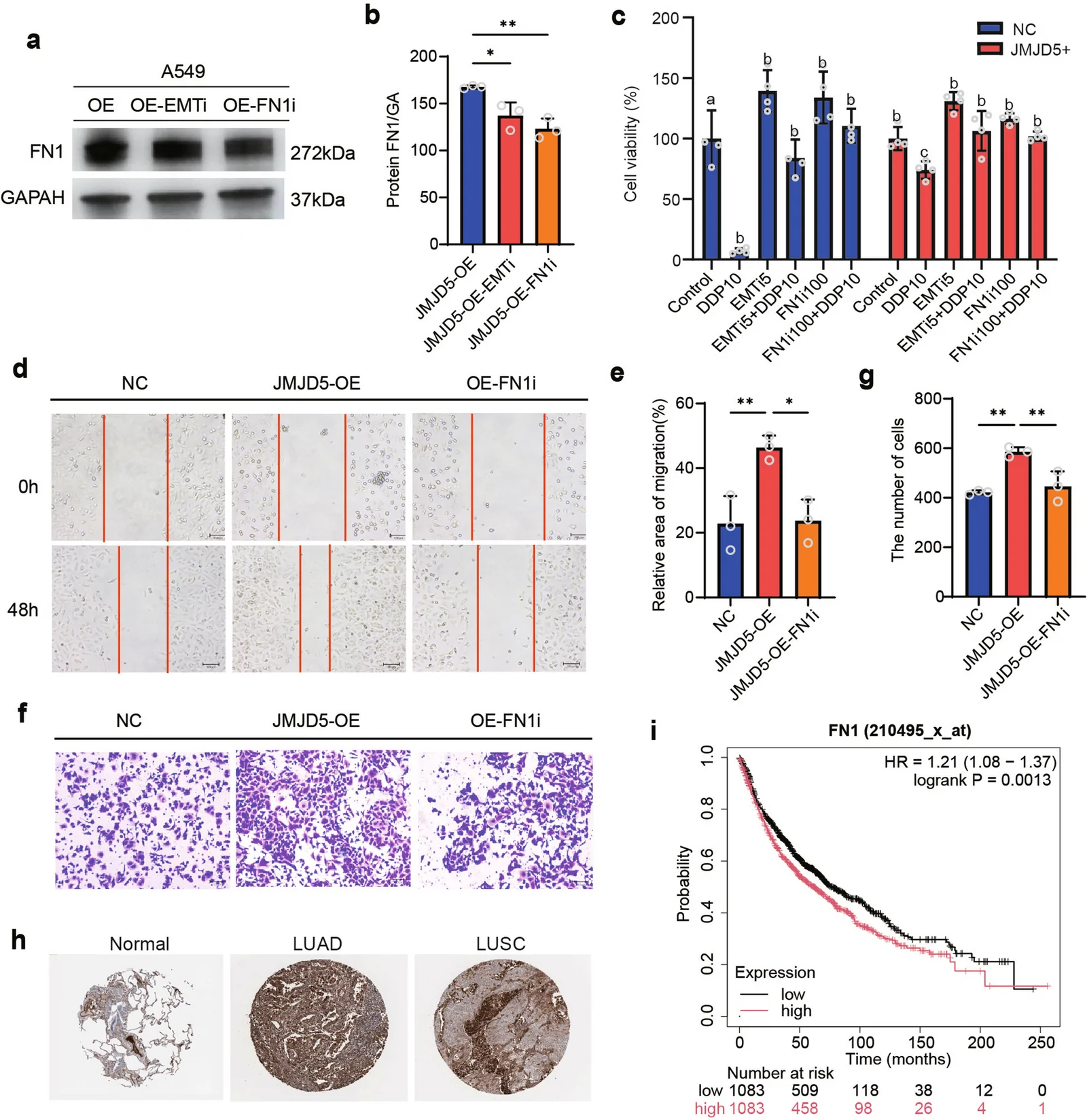
Pharmacological inhibition of FN1 reverses JMJD5-induced malignant phenotypes and cisplatin resistance, and FN1 expression is associated with poor prognosis in lung cancer. **a**, **b** Representative western blot images showing FN1 protein expression in control and JMJD5-overexpressing A549 cells treated with an EMT inhibitor (EMTi) or FN1 inhibitor (FN1i) (**a**), with densitometric quantification normalized to the loading control (**b**). **c** CCK-8 assay evaluating the effects of EMTi and FN1i on cell viability following cisplatin treatment in A549^NC^ and A549.^JMJD5+^ cells. Letter annotations indicate statistical differences among groups: “a” denotes a significant difference from all other groups, “b” denotes a significant difference from at least one other group, and “c” denotes no significant difference from the other groups. **d**, **e** Representative wound-healing images showing the effect of FN1 inhibition on the migratory capacity of JMJD5-overexpressing A549 cells (**d**), with quantification of wound closure (**e**), Scale bar, 100 μm. **f**, **g** Representative Transwell invasion images showing the effect of FN1 inhibition on the invasive capacity of JMJD5-overexpressing A549 cells (**f**), with quantification of invaded cells (**g**), Scale bar, 100 μm. **h** Representative HPA immunohistochemical images showing FN1 expression in normal lung, lung adenocarcinoma, and lung squamous cell carcinoma tissues, data are sourced from the Human Protein Atlas (https://www.proteinatlas.org/). **i** Kaplan–Meier analysis showing that high FN1 expression is associated with shorter overall survival in patients with lung cancer (HR = 1.21, 95% CI: 1.08–1.37; log-rank *P* = 0.0013). All experiments were repeated three times. Data are presented as mean ± SD. *P < 0.05, **P < 0.01, ***P < 0.001 and ****P < 0.0001

To further establish the functional role of FN1 in JMJD5-mediated cisplatin resistance, JMJD5-overexpressing A549 cells were treated with an FN1 inhibitor or an EMT inhibitor (EMTi). Both interventions significantly reduced FN1 protein levels (Fig. 7a-b, *P* < 0.05). CCK-8 assays showed that JMJD5 overexpression increased cell viability following cisplatin exposure, whereas inhibition of either FN1 or EMT significantly restored cisplatin sensitivity (Fig. 7c). FN1 inhibition also attenuated JMJD5-induced cell migration and invasion, as demonstrated by wound-healing and Transwell assays (Fig. 7d-g; *P* < 0.05).

The clinical relevance of FN1 was further assessed using HPA immunohistochemistry and Kaplan–Meier survival analysis. FN1 showed cell type-specific expression in normal lung tissue, with moderate staining in alveolar type II cells and macrophages but little or no staining in alveolar type I or endothelial cells. Representative IHC images showing FN1-positive staining are presented in Fig. 7h. Moreover, high FN1 expression was significantly associated with shorter overall survival in patients with lung cancer (HR = 1.21, 95% CI: 1.08–1.37; log-rank *P* = 0.0013; Fig. 7i).

Together, these findings support FN1 as a functional downstream mediator of JMJD5-associated EMT phenotypes and cisplatin resistance and further indicate its clinical relevance in lung cancer.

### JMJD5-dependent FN1 protein accumulation requires ongoing protein synthesis

**Fig. 8:**
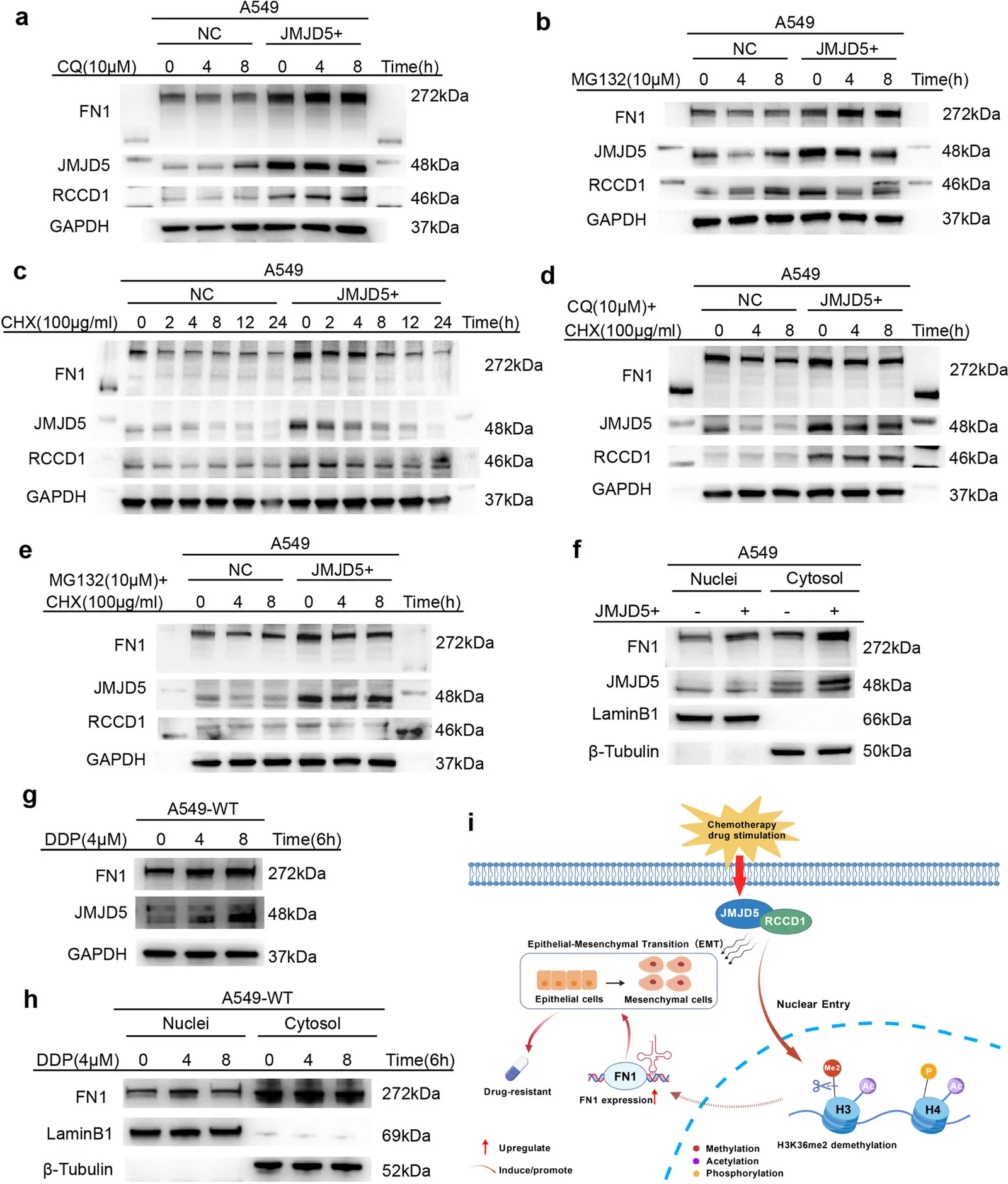
JMJD5 enhances FN1 protein synthesis and promotes cisplatin resistance in NSCLC cells. **a** Western blot analysis of FN1 protein accumulation in A549^NC^ and A549^JMJD5+^ cells following inhibition of lysosomal degradation with chloroquine (CQ). **b** Western blot analysis of FN1 protein accumulation in A549^NC^ and A549^JMJD5+^ cells following proteasomal inhibition with MG132. **c** Western blot analysis of FN1 protein levels in A549^NC^ and A549^JMJD5+^ cells following cycloheximide (CHX) treatment. **d** Western blot analysis of FN1 protein levels in A549^NC^ and A549^JMJD5+^ cells following combined CHX and CQ treatment. **e** Western blot analysis of FN1 protein levels in A549^NC^ and A549^JMJD5+^ cells following combined MG132 and CHX treatment. **f** Western blot analysis of the subcellular distribution of FN1 in cytosol and nuclei following JMJD5 overexpression. **g** Western blot analysis showing increased JMJD5 and FN1 expression in A549 cells following cisplatin (DDP) treatment. **h** Western blot analysis of nuclear and cytoplasmic FN1 expression in A549 cells following 6 h of DDP treatment. **i** Schematic model illustrating the proposed JMJD5–RCCD1–FN1 regulatory axis, whereby JMJD5 interacts with RCCD1 to facilitate H3K36me2 demethylation, enhance FN1 expression, and promote EMT-associated platinum resistance in NSCLC. NC, negative control; JMJD5 +, JMJD5-overexpressing cells; CQ, chloroquine; CHX, cycloheximide; DDP, cisplatin. The schematic was created using BioGDP.com

Since JMJD5 significantly promotes FN1 protein expression(Fig. 6a), yet does not significantly affect the mRNA level of FN1 (Fig. S2f), we further examined whether JMJD5 affects the protein degradation of FN1 through the proteasome or lysosome pathway using the lysosomal inhibitor chloroquine (CQ) and the proteasome inhibitor MG132. We also utilized the translation elongation inhibitor cycloheximide (CHX) to assess its role in the protein synthesis of FN1. The results showed that JMJD5 accumulated after CQ treatment in A549^NC^ cells and in A549^JMJD5+^ cells, accompanied by the accumulation of RCCD1 in A549^JMJD5+^ cells (Fig. 8a). Notably, FN1 was significantly accumulated in JMJD5-overexpressed cells (A549^JMJD5+^ cells), but the accumulation of JMJD5 in A549^NC^ cells did not contribute to FN1 accumulation. Similar accumulation of FN1 was observed in the MG132-treated group, although JMJD5 and RCCD1 accumulation did not show comparable accumulation in A549^JMJD5+^ cells (Fig. 8b). However, CHX treatment blocked the increase in FN1 expression upon JMJD5 overexpression (Fig. 8c). This blockade persisted regardless of whether the lysosome or proteasome pathways were inhibited (Fig. 8d-e). These findings indicate that the JMJD5-associated increase in FN1 protein abundance requires ongoing protein synthesis and is unlikely to be explained primarily by altered lysosomal or proteasomal degradation. However, whether JMJD5 directly enhances FN1 translation remains to be determined.

We further investigated whether JMJD5 differentially regulates intracellular versus extracellular FN1 expression, and whether cisplatin induces nucleocytoplasmic redistribution of FN1. The results showed that JMJD5 overexpression upregulated FN1 levels in both the nucleus and the cytoplasm (Fig. 8f). DDP stimulation upregulated JMJD5 and total FN1 protein levels in A549 cells (Fig. 8g, Fig. S2h), and also altered the nuclear–cytoplasmic distribution of FN1 (Fig. 8h, Fig. S2i). Taken together, our results suggest that JMJD5 promotes the total synthesis of FN1 protein without affecting the subcellular localization dynamics of FN1.

Therefore, our study suggests that JMJD5 exerts its demethylase activity by interacting with the partner protein RCCD1, which promotes the translation of the downstream target fibronectin FN1. This process mediates the formation of a mesenchymal-like phenotype in NSCLC, thereby leading to platinum resistance in NSCLC (Fig. 8i).

## Discussion

Our previous clinical study demonstrated that elevated JMJD5 expression was associated with unfavorable survival in patients with NSCLC receiving platinum-based chemotherapy [4], suggesting a potential role for JMJD5 in treatment resistance. Here, we provide functional and mechanistic evidence supporting this association. JMJD5 overexpression reduced the sensitivity of NSCLC cells to cisplatin and carboplatin in vitro and impaired the antitumor efficacy of cisplatin in vivo, whereas no comparable resistance was observed with pemetrexed or paclitaxel. Unexpectedly, JMJD5 simultaneously suppressed cell proliferation and clonogenic growth, consistent with a previous report showing an antiproliferative effect of JMJD5 in p53-wild-type NSCLC cells [18]. This apparent paradox may reflect a phenotypic trade-off between proliferation and stress tolerance. EMT and cancer stem-like states are widely implicated in therapeutic resistance [19, 20], and acquisition of stem-like properties may be accompanied by reduced proliferative activity under basal conditions [21, 22]. Accordingly, JMJD5-overexpressing cells displayed reduced proliferative fitness but increased spheroid formation, migration, invasion, and resistance to platinum-induced cytotoxicity.

These findings support a model in which JMJD5 promotes a relatively slow-cycling, mesenchymal/stem-like state that favors survival under chemotherapy. EMT has been linked to chemoresistance in both pancreatic [23] and lung cancer [24], where mesenchymal tumor cells may exhibit reduced proliferation, enhanced resistance to apoptosis, and increased expression of drug-resistance programs [23, 25]. Consistently, JMJD5 overexpression decreased E-cadherin while increasing N-cadherin and vimentin, whereas JMJD5 depletion produced the opposite effects. EMT inhibition partially reversed these changes without altering JMJD5 expression, placing JMJD5 upstream of the EMT program. This interpretation is also consistent with previous evidence linking JMJD5 to cancer stem cell-like phenotypes [26]. Thus, the enhanced platinum resistance observed despite slower tumor growth likely reflects phenotypic adaptation rather than increased proliferative capacity.

As a member of the JmjC domain-containing epigenetic regulator family, JMJD5 has been reported to function as a histone demethylase [5], an arginine hydroxylase [6] and a Cathepsin L-type protease [7–9]. Our data show that cisplatin increased JMJD5 expression and nuclear accumulation concomitant with reduced H3K36me2 levels, implicating JMJD5-associated chromatin remodeling in chemotherapy-induced stress adaptation. Because JMJD5 lacks a canonical histone-binding domain [17], we investigated potential interacting partners and identified RCCD1 by IP–MS and Co-IP. Importantly, RCCD1 depletion attenuated the JMJD5-associated reduction in H3K36me2 and reversed JMJD5-induced migration, invasion, EMT-associated changes, and cisplatin resistance. These findings support RCCD1 as a functional cofactor required for JMJD5-mediated chromatin remodeling rather than merely a binding partner.

RCCD1 has previously been implicated in EMT-related tumor progression [27–29], and RCCD1 depletion has been reported to stabilize cytoskeletal microtubules and attenuate TGF-β-induced EMT and migration [28]. Given the central role of TGF-β signaling in EMT regulation [30], RCCD1 may represent a convergence point between extracellular EMT-inducing signals and JMJD5-associated epigenetic remodeling. In our model, cisplatin promoted the nuclear accumulation of both JMJD5 and RCCD1, coinciding with H3K36me2 reduction. This coordinated response suggests that chemotherapy-induced stress activates a JMJD5–RCCD1-dependent chromatin state that facilitates acquisition of a drug-tolerant mesenchymal phenotype.

JMJD5 has also been implicated in transcriptional regulation through histone-tail processing and facilitation of RNA polymerase II progression [7, 8]. Screening of JMJD5-dependent downstream targets identified FN1 as a prominent functional effector. FN1 is a major extracellular matrix glycoprotein involved in cell adhesion, migration, and differentiation [31], and increased FN1 expression has been linked to chemoresistance [32] and EMT-associated tumor progression [33]. In the present study, genetic depletion of FN1 suppressed JMJD5-induced migration, invasion, EMT-associated changes, and cisplatin resistance. Importantly, pharmacological inhibition of FN1 reproduced these effects, further supporting a functional requirement for FN1 in the JMJD5-driven resistant phenotype. The clinical relevance of these findings was supported by representative HPA immunohistochemistry showing FN1 expression in lung cancer cells and by Kaplan–Meier analysis demonstrating significantly shorter overall survival in patients with high FN1 expression (HR = 1.21, 95% CI: 1.08–1.37; log-rank *P* = 0.0013). Together, these observations support FN1 as an important downstream mediator connecting JMJD5–RCCD1 activity with EMT-associated malignant behavior and platinum resistance.

Notably, our data do not support a simple model in which JMJD5–RCCD1-mediated H3K36me2 demethylation directly activates FN1 transcription. Although FN1 protein abundance increased markedly following JMJD5 overexpression, no significant commensurate elevation in FN1 mRNA was observed in our PCR‑based validation assays. FN1 has been reported to undergo p62-dependent autophagy–lysosomal degradation rather than predominant ubiquitin–proteasome-mediated turnover [34]. However, inhibition of either lysosomal or proteasomal degradation did not account for the JMJD5-dependent increase in FN1, whereas cycloheximide abolished FN1 accumulation even when these degradation pathways were inhibited. Thus, increased FN1 abundance requires ongoing protein synthesis, but whether JMJD5 directly enhances FN1 translation remains unresolved. A more plausible interpretation is that JMJD5–RCCD1-dependent chromatin remodeling influences intermediate transcriptional, post-transcriptional, or translational regulators that ultimately control FN1 protein production.

Several limitations should be acknowledged. First, although public ChIP-seq datasets suggest H3K36me2 enrichment at the FN1 genomic locus, our study did not include direct assessment of JMJD5/RCCD1 occupancy or locus-specific H3K36me2 remodeling. Future validation by ChIP-qPCR, CUT&RUN, or related approaches would further strengthen the evidence for this regulatory relationship. Second, the molecular intermediates linking JMJD5–RCCD1-dependent chromatin remodeling to FN1 protein synthesis remain unknown. Third, although genetic and pharmacological rescue experiments support an important contribution of FN1 and EMT to cisplatin resistance, additional EMT-independent mechanisms cannot be excluded. Fourth, the IHC analysis was based on representative HPA specimens and different antibodies were used for normal and tumor tissues; moreover, the survival analysis was unadjusted. Validation of the coordinated JMJD5/RCCD1/H3K36me2/FN1 phenotype in well-annotated, platinum-treated NSCLC cohorts is therefore warranted. Finally, the mechanism underlying cisplatin-induced nuclear accumulation of JMJD5 and RCCD1 requires further investigation, as does the in vivo relevance of JMJD5 in lung squamous cell carcinoma.

In summary, our findings support a model in which cisplatin-induced JMJD5–RCCD1 activity promotes H3K36me2 remodeling and a stress-tolerant mesenchymal phenotype, with FN1 acting as an important downstream functional effector. Disruption of RCCD1 or FN1 restores cisplatin sensitivity and attenuates malignant phenotypes, identifying the JMJD5–RCCD1–FN1 axis as a potential therapeutic vulnerability in platinum-resistant NSCLC while highlighting key mechanistic links that remain to be resolved.

## Conclusion

In conclusion, our study identifies RCCD1 as an important functional partner of JMJD5 in H3K36me2 remodeling and demonstrates that JMJD5/RCCD1 activity contributes to mesenchymal-like phenotypes and platinum resistance in NSCLC. FN1 acts as an important downstream functional effector associated with these phenotypes, although the molecular link connecting JMJD5–RCCD1-dependent chromatin remodeling to FN1 protein regulation remains to be fully elucidated.

## Methods and materials

### Cell culture

Human NSCLC cell lines A549 and H2170 cells (obtained from Meisen CTCC, China) were cultured in RPMI 1640 medium (Cat# SH30809.01, Cytiva, USA) supplemented with 10% fetal bovine serum (Cat# 10099–141, GIBCO, USA) and 1% streptomycin/penicillin (Cat# CR15140, Cienry, Zhejiang) in RPMI at a temperature of 37℃ and a CO_2_ concentration of 5%.

### CRISPR/Cas9, SiRNAs, and lentiviral infection

CRISPR/Cas9-mediated knockout of JMJD5 or RCCD1 was generated using the pLenti-U6-sgRNA-SFFV-Cas9-2A-Puro vector, while JMJD5 overexpression was achieved via lentiviral delivery of the pLenti-CMV-CBH-Puro Amp vector containing FLAG/GFP-tagged constructs. Polybrene (Cat# GC19206, GLPBIO, USA) was added to the culture medium for at least 48 h to enhance infection efficiency. Puromycin (Cat# ST551, Beyotime, China) selection (1 μg/mL, 14 days) was applied to obtain stable polyclonal cells, with successful modifications confirmed by Western blot and sequencing.

Small interfering RNA (siRNA) was also used for gene silencing. Scrambled siRNA and siRNAs targeting JMJD5 or FN1 were purchased from Genepharma (Shanghai, China). The siRNA sequences used in this study are provided in Table S1. Transfection was performed using Lipofectamine 2000 (Cat# CN2541156, Invitrogen, USA) in serum-free Opti-MEM (Cat# 2898893, Gibco, USA), following the manufacturer's protocol. The transfection medium was replaced with complete growth medium 6 h post-transfection.

### Western Blot and antibodies

Western blot was performed as previously reported [35]. All antibodies used in this study are listed below: JMJD5 (Cat# ab36104, Abcam, UK), JMJD5 (Cat# PA5-8816, Invitrogen, USA), RCCD1(Cat# abs153101, Absin, China), FN1 (Cat# ET1702-25, HUABIO, China), E-cadherin (Cat# 20874-I-AP, Proteintech, China), N-cadherin (Cat# 22018–1-AP, Proteintech, China), Vimentin (Cat# 10366-I-AP, Proteintech, China), β-tubulin (Cat# ET1602-4, HUABIO, China), Lamin B1 (Cat# ET160627, HUABIO, China), H3K36me2 (Cat# DF6943, Affinity, USA), GAPDH (Cat# db11729, diagbio, China), H3 (Cat# ET1701-64, HUABIO, China). Cytoplasmic and nuclear proteins were isolated using the NE-PER™ Nuclear and Cytoplasmic Extraction Reagents (Cat# 78833, Invitrogen, USA).

### RT-qPCR and PCR array

Total RNA was extracted using the RNA Rapid Extraction Kit (Cat#RN001, ES Science, China). The RNA concentration was measured and quantified, followed by reverse transcription into cDNA. RT-qPCR was carried out with TB Green™ Premix Ex Taq™ (Cat# RR420A, Takara, Japan). Detailed primer sequences can be found in Table S2.

### Cell viability assay

Cells were seeded in 96-well plates at a density of 3000 cells/well. For cell growth detection, cell viability was assessed using the CCK-8 assay (Cat# GK10001, GLPBIO, USA) after 48 h, with a measurement wavelength of 450 nm.

### Drug sensitivity assay

A549 or H2170 cells from control and experimental groups (gene knockout or overexpression) were seeded at a density of 3,000 cells per well in 96-well plates. Twenty-four hours post-seeding, cells were treated with chemotherapy drugs, including carboplatin (CBP) (Cat: H20020181, qilu), cisplatin (DDP)(Cat: X20010105, haosen), Paclitaxel Injection (PTX)(purchased from Cisen), and pemetrexed disodium (PBM)(purchased from qilu). After 48 h of drug exposure, the medium containing the drugs was removed. A blank medium mixed with CCK-8 reagent in a 100:10 ratio was then added to each well. The plates were incubated for 1–3 h, after which cell viability was assessed.

For all drug sensitivity assays in this study, a wide-range logarithmic concentration gradient was first applied to estimate the IC₅₀ range. Once confirmed, a narrower concentration gradient was used for detailed sensitivity testing. All experiments were performed in triplicate, with each concentration tested in at least three replicate wells.

### Colony formation assay

Cells were seeded in 6-well plates at a density of 500 cells per well. Following 1–2 weeks of culture, cell colonies were fixed with 4% paraformaldehyde for 15–30 min and then stained with crystal violet for 15–30 min for visualization and quantification.

### Wound healing assay

A wound healing assay was performed to assess cell migration. Briefly, Cells were evenly seeded in 12-well plates and cultured to full confluence. A uniform scratch was made in each well using a sterile pipette tip. The wells were then rinsed 3–4 times with PBS to remove detached cells, and images were captured under a microscope at 0 h and 48 h.

### Transwell assay

Cells were seeded in the upper chambers of the Transwell apparatus (Cat# CN-3422, corning, China) at a density of 2 × 10^4^ cells per well, with medium containing 2% FBS. The lower chambers were filled with complete medium. After 48 h of incubation, the migrated cells were fixed with 4% paraformaldehyde, stained with crystal violet for visualization and quantification.

### Sphere formation assay

Cells were seeded a density of in ultra-low attachment 24-well plates (Cat# 3473, corning, China) at a density of 500 cells per well. The cells were cultured in stem cell suspension medium, which consisted of DMEM/F12 (Cat# SH30023.01, Cytiva, USA), 2% B27 (Cat# 17504044, GIBCO, USA), 20 ng/mL bFGF (Cat# AF-100-18B-50, Guangzhou Miaobo, China), 10 ng/ml EGF (Cat# HY-P7109, MCE, USA), 5 μg/ml insulin (Cat# 11061–68-0, Shanghai Taoshu, China), and penicillin–streptomycin (Cat# CR15140, Senrui Biotechnology, China). After 14 days of culture, the number and size of tumorspheres were examined under an optical microscope.

### Co-immunoprecipitation (Co-IP) analysis

Cell lysates were prepared using IP lysis (Cat# P0013, Beyotime, China) buffer supplemented with protease and phosphatase inhibitors(Cat# ST507-10 ml, Beyotime, China). Specific antibodies (JMJD5, Cat# sc-377078, Absin, China; IgG, Cat# A7028, Beyotime, China) were added to each group and incubated overnight at 4 °C. Subsequently, Protein A/G Agarose (Cat# P2055, Beyotime, China) was added, and the mixture was incubated at 4 °C for 4 h with gentle shaking. After centrifugation at 1000 g, the supernatant was discarded, and the beads were washed with wash buffer. Finally, the samples were resuspended, boiled, and then collected for gel analysis.

### Immunofluorescence

After 6 h of DDP stimulation, the cells were fixed with 4% formaldehyde, permeabilized with 0.5% Triton X-100 and blocked with 5% BSA. The cells were then incubated with primary antibodies against JMJD5 and RCCD1, followed by treatment with specific fluorescent secondary antibodies (Anti-mouse, Cat# SA00013-3, Proteintech, China; Anti-rabbit, Cat# SA00013-2, Proteintech, China) for 1 h. The cells were also stained with DAPI (Cat# C1005, Beyotime, China) for nuclei visualization. Images were acquired using a confocal laser scanning microscope (Carl Zeiss, LSM780, Oberkochen, Germany) under oil immersion and analyzed using the FV10-ASW 4.2 Viewer software.

### Xenograft model

JMJD5-overexpressing A549 cells (A549^JMJD5+^) and the control cells (A549^NC^) were suspended in serum-free medium at a density of 5 × 10^6^ cells/100 μl per mouse. The cell suspension was injected subcutaneously into 4–6-week-old male BALB/c nude mice (3 mice per group). After 10 days, tumor tissues were surgically resected, dissected into 2 × 2 × 2 mm^3^ blocks, and subcutaneously implanted into 24 BALB/c mice (12 mice per group). The tumor volume was calculated using the standard formula: (length × width ^2^)/2. When the average tumor volume reached 100–200 mm^3^(approximately 2 weeks later), the 12 mice in each group were further randomized into two subgroups (1:1 ratio), with 6 mice assigned to each treatment group. Cisplatin (6 mg/kg) or 0.9% saline solution was then administered every three days via tail vein injection (*n* = 6).

Tumor size, body weight, and general condition were monitored every 3 days throughout the treatment period. Tumor volume was calculated using the formula: (length × width^2^)/2. Although the cisplatin dose was selected with reference to previously published experimental protocols [36], reduced treatment tolerance and deterioration in general condition were observed during repeated administration in the present model. To minimize excessive treatment-related toxicity and avoid potential mortality, the in vivo experiment was terminated earlier than originally planned based on animal welfare considerations, and xenograft tumors were subsequently harvested and weighed. Consequently, the duration of tumor-growth observation was limited, resulting in relatively small terminal tumor volumes. The animal study was approved by the Laboratory Animal Ethics Committee of Zhejiang Cancer Hospital (No. zjzlsd-2021–04-002).

### Pharmacological rescue experiments

To evaluate whether EMT and FN1 signaling are required for JMJD5-mediated cisplatin resistance and malignant phenotypes, pharmacological rescue experiments were performed using an epithelial–mesenchymal transition inhibitor (EMTi) (CAS: 1638526–21-2) and an Fibronectin Inhibitor (FN1i) (CAS: 91037–65-9). For functional assays, EMTi was applied at 5, 10, or 20 μM and FN1i was used at 100 μM. Cisplatin (DDP) was used at 10 μM, and co-treatment with DDP and inhibitors was performed for indicated time periods. For cell viability analysis, A549 cells stably expressing JMJD5 or control vector were seeded in 96-well plates at a density of 3 × 10^3^ cells per well and treated with DDP in the presence or absence of EMTi or FN1i, followed by CCK-8 measurement at 24, 48, and 72 h according to the manufacturer’s instructions. For migration and invasion assays, cells were treated with EMTi (5 μM) or FN1i (100 μM) during the experimental period; migration was assessed using a wound-healing assay with imaging at 0, 24, 48, and 72 h after scratching, and invasive capacity was evaluated using a Transwell system in which 1–2 × 10^4^ cells were seeded into the upper chamber and allowed to invade for 24 h, followed by crystal violet staining and quantification of invaded cells. All experiments were independently repeated at least three times.

### Analysis of H3K36me2 enrichment at RCCD1 locus and FN1 promoter

We retrieved genome browser tracks from the Cistrome database (https://db3.cistrome.org/browser/) to investigate whether the RCCD1 (chr15:90,953,884–90,964,125) genomic locus is enriched for H3K36me2 histone modifications. We further examined the promoter region of FN1 (chr2: 215.36–215.44 Mb (hg38)) for significant H3K36me2 peak enrichment. These analyses were performed to assess the potential association between RCCD1 and H3K36me2-mediated chromatin regulation, and to predict FN1 as a putative epigenetic target potentially regulated through H3K36me2-dependent chromatin remodeling.

### FN1 expression and survival analysis

Immunohistochemical (IHC) expression patterns of FN1 in normal lung and lung cancer tissues were obtained from the Human Protein Atlas (HPA) [37]. The HPA content was used in accordance with the Creative Commons Attribution 4.0 International (CC BY 4.0) License. Representative images were appropriately credited to the Human Protein Atlas in the corresponding figure legend. Normal lung tissues were stained with the HPA027066 antibody, whereas lung adenocarcinoma and lung squamous cell carcinoma tissues were stained with the CAB000126 antibody. Because different antibodies were used for normal and tumor tissues, comparisons between these groups were restricted to qualitative assessment of FN1 expression patterns. The association between FN1 expression and overall survival (OS) in patients with lung cancer was further evaluated using the Kaplan–Meier Plotter platform (https://kmplot.com/analysis/). Patients were stratified into high- and low-expression groups according to the median FN1 expression level. Survival differences were assessed using the log-rank test, and hazard ratios (HRs) with 95% confidence intervals (CIs) were calculated.

### Statistical analysis

Quantitative analysis of image data was performed using ImageJ software, while data analysis and graphing were conducted using GraphPad Prism 9. Statistical analysis was carried out using the t-test, one-way ANOVA, and Two-way ANOVA. Data are presented as mean ± SD. *P* < 0.05 was found to be statistically significant. The significance thresholds were defined as follows: ns (not significant) for *P* > 0.05, *P < 0.05, **P < 0.01, ***P < 0.001, and ****P < 0.0001. Except for animal experiments, all other experiments were independently repeated three times for biological reproducibility.

## Supplementary Information

Supplementary Material 1. 

Supplementary Material 2. Table S3. Co-immunoprecipitation coupled with mass spectrometry (Co-IP/MS) analysis of A549 cells transfected with NC or JMJD5-overexpressing.

Supplementary Material 3. Table S4. Identification of candidate downstream stemness-associated targets by stem cell-focused PCR array in NC and JMJD5-overexpressing cells.

## Data Availability

The datasets generated during and/or analysed during the current study are not publicly available due to [Part of the research project has not been completed.] but are available from the corresponding author on reasonable request.
