# Supplementary material for: RCCD1-JMJD5 coordinate histone demethylation to promote platinum resistance in non-small cell lung cancer via FN1-mediated epithelial–mesenchymal transition
Source: Mol Biomed. 2026 Oct 21;7:222. doi: 10.1186/s43556-026-00625-y (PMC13687518; doi:10.1186/s43556-026-00625-y)
Supplement: Supplementary file 1 [file 43556_2026_625_MOESM1_ESM.docx]

**RCCD1-JMJD5 Coordinate Histone Demethylation to Promote Platinum Resistance in Non-Small Cell Lung Cancer via FN1-Mediated Epithelial–Mesenchymal Transition**

Lili Tan^1,2,#^, Yinghui Tong^1,#^, Wanying Zhou^1,2^, Xinyue Ma^1,2^, Qilu Fang^1^, Yan Feng^1^, Xueping Xiang^3,*^, Luo Fang^1,2,*^

^1^Zhejiang Cancer Hospital, Hangzhou Institute of Medicine (HIM), Chinese Academy of Sciences, Hangzhou, Zhejiang 310022, China.

^2^Postgraduate·Training Base Alliance of Wenzhou Medical University·(Zhejiang Cancer Hospital),·Hangzhou, Zhejiang, 310022, China.

^3^Department of Pathology, the Second Affiliated hospital, School of Medicine, Zhejiang University, Hangzhou 310009, China.

^#^Lili Tan and Yinghui Tong contributed equally to this work.

*Correspondence:

Luo Fang: E-mail: [fangluo@zjcc.org.cn](mailto:fangluo@zjcc.org.cn);

Xueping Xiang: Email: xiangxueping@zju.edu.cn.

**Fig. S1 JMJD5 modulates chemotherapeutic response and interacts with RCCD1 in lung adenocarcinoma cells. a, b** CCK-8 assays showing the viability of A549^NC^ and A549^JMJD5+^ cells following 48 h treatment with increasing concentrations of pemetrexed (PBM) (**a**) or paclitaxel (PTX) (**b**). **c** Representative images of subcutaneous xenograft tumors harvested from BALB/c nude mice. **d, e** Quantitative analysis of xenograft tumor volumes derived from A549^NC^ and A549^JMJD5+^ cells following treatment with normal saline or cisplatin (DDP). **f, g** Quantification of JMJD5 (**f**) and H3K36me2 (**g**) protein levels in A549 wild-type (A549^WT^) cells following treatment with increasing concentrations of DDP (0, 4, and 8 μM). **h** Endogenous co-immunoprecipitation (Co-IP) assay validating the interaction between JMJD5 and RCCD1 identified by immunoprecipitation–mass spectrometry (IP–MS). NC, negative control; JMJD5+, JMJD5-overexpressing cells; WT, wild type; PBM, pemetrexed; PTX, paclitaxel; DDP, cisplatin. All experiments were repeated three times. Data are presented as mean ± SD. *P < 0.05, **P < 0.01, ***P < 0.001 and ****P < 0.0001.

**Fig. S2 Cisplatin promotes nuclear accumulation of JMJD5/RCCD1 and induces FN1 expression in lung adenocarcinoma cells.** **a** Quantification of RCCD1 protein levels in A549 wild-type (A549^WT^) cells following treatment with increasing concentrations of cisplatin (DDP; 0, 4, and 8 μM). **b, c** Quantification of nuclear and cytoplasmic JMJD5 (**b**) and RCCD1 (**c**) protein levels in A549^NC^ and A549^JMJD5+^ cells following treatment with increasing concentrations of DDP (0, 4, and 8 μM). **d** Quantification of nuclear and cytoplasmic H3K36me2 levels in A549^NC^ and A549^JMJD5+^ cells following DDP treatment. **e** Identification of candidate downstream stemness-associated genes exhibiting >5-fold differential expression in A549^JMJD5+^ cells using a stem cell-related PCR array. **f** qRT-PCR validation of selected candidate genes in A549^NC^ and A549^JMJD5+^ cells. **g** Quantification of FN1 protein levels in A549WT cells following treatment with increasing concentrations of DDP (0, 4, and 8 μM). **h** Quantification of nuclear and cytoplasmic FN1 protein levels in A549^NC^ and A549^JMJD5+^ cells following DDP treatment. NC, negative control; JMJD5+, JMJD5-overexpressing cells; WT, wild type; DDP, cisplatin. For letter-based statistical annotations, “a” indicates a significant difference from all comparison groups, “b” indicates a significant difference from at least one comparison group, and “c” indicates no significant difference from any comparison group. Data are presented as mean ± SD from three independent experiments. *P < 0.05, **P < 0.01, ***P < 0.001 and ****P < 0.0001.

**Table S1. siRNA sequences**

| Gene Name（human） | Sequences | | Gene ID |
| --- | --- | --- | --- |
|  | sense（5'-3'） | antisense（5'-3'） |  |
| KDM8-Homo-544 | GGGACAUCCUUCUUAAAGUTT | ACUUUAAGAAGGAUGUCCCTT | 79831 |
| KDM8-Homo-662 | GAUUCCAGAUGUGAAGUUATT | UAACUUCACAUCUGGAAUCTT | 79831 |
| KDM8-Homo-220 | CGCACAGUAAAGAAGACCUTT | AGGUCUUCUUUACUGUGCGTT | 79831 |
| KDM8-Homo-919 | GCAAAUACAUCGUGAAUGATT | UCAUUCACGAUGUAUUUGCTT | 79831 |
| FN1-Homo-411 | GCCCGGUUGUUAUGACAAUTT | AUUGUCAUAACAACCGGGCTT | 2335 |
| FN1-Homo-2093 | GUCCUGUCGAAGUAUUUAUTT | AUAAAUACUUCGACAGGACTT | 2335 |
| FN1-Homo-5036 | GUCCAGAUCAAACAGAAAUTT | AUUUCUGUUUGAUCUGGACTT | 2335 |
| FN1-Homo-6056 | CCGGCUACAUCAUCAAGUATT | UACUUGAUGAUGUAGCCGGTT | 2335 |

**Table S2. PCR Primer RNA sequences**

| Primer | sense（5'-3'） | antisense（5'-3'） |
| --- | --- | --- |
| CCL7 | CCCTGGGAAGCTGTTATCTTCAA | CTCGACCCACTTCTGATGGG |
| SSTR2 | TGGCTATCCATTCCATTTGACC | AGGACTGCATTGCTTGTCAGG |
| FN1 | AGGAAGCCGAGGTTTTAACTG | AGGACGCTCATAAGTGTCACC |
| JMJD5-1 | CCATCAATGCCTGGTTTGGTC | GTGCGTGTCATGAGGGTACAGAG |
| JMJD5-2 | CGTGCATGCAGAAGTGGAGT | TACCCGACGTCCCTTGGCTC |
